# MRI Findings in 77 Children with Non-Syndromic Autistic Disorder

**DOI:** 10.1371/journal.pone.0004415

**Published:** 2009-02-10

**Authors:** Nathalie Boddaert, Mônica Zilbovicius, Anne Philipe, Laurence Robel, Marie Bourgeois, Catherine Barthélemy, David Seidenwurm, Isabelle Meresse, Laurence Laurier, Isabelle Desguerre, Nadia Bahi-Buisson, Francis Brunelle, Arnold Munnich, Yves Samson, Marie-Christine Mouren, Nadia Chabane

**Affiliations:** 1 INSERM-CEA U 797, Service Hospitalier Frédéric Joliot, CEA, Orsay, France; 2 Service de Radiologie Pédiatrique, U 797, Hôpital Necker Enfants Malades, AP-HP, Paris V, Paris, France; 3 Service de Pédopsychiatrie, Hôpital Robert Debré, AP-HP, Paris, France; 4 Genetic Clinic and Research Unit INSERM 781, Hôpital Necker-Enfants Malades and Université Paris V René Descartes, Paris, France; 5 Service de Pédopsychiatrie, Hôpital Necker Enfants Malades, AP-HP, Paris V, Paris, France; 6 Service de Neurologie et Métabolisme, Hôpital Necker Enfants Malades, AP-HP, Paris V, Paris, France; 7 INSERM U 916, CHU Bretonneau, Tours, France; 8 Service des Urgences Cerebro-Vasculaires, Groupe Hospitalier Pitié-Salpêtrière, AP-HP, Paris, France; James Cook University, Australia

## Abstract

**Background:**

The clinical relevance of MR scanning in children with autism is still an open question and must be considered in light of the evolution of this technology. MRI was judged to be of insufficient value to be included in the standard clinical evaluation of autism according to the guidelines of the American Academy of Neurology and Child Neurology Society in 2000 [1]. However, this statement was based on results obtained from small samples of patients and, more importantly, included mostly insufficient MRI sequences. Our main objective was to evaluate the prevalence of brain abnormalities in a large group of children with a non-syndromic autistic disorder (AD) using T1, T2 and FLAIR MRI sequences.

**Methodology:**

MRI inspection of 77 children and adolescents with non-syndromic AD (mean age 7.4±3.6) was performed. All met the DSM-IV and ADI –R criteria for autism. Based on recommended clinical and biological screenings, we excluded patients with infectious, metabolic or genetic diseases, seizures or any other neurological symptoms. Identical MRI inspections of 77 children (mean age 7.0±4.2) without AD, developmental or neurological disorders were also performed. All MRIs were acquired with a 1.5-T Signa GE (3-D T1-FSPGR, T2, FLAIR coronal and axial sequences). Two neuroradiologists independently inspected cortical and sub-cortical regions. MRIs were reported to be normal, abnormal or uninterpretable.

**Principal Findings:**

MRIs were judged as uninterpretable in 10% (8/77) of the cases. In 48% of the children (33/69 patients), abnormalities were reported. Three predominant abnormalities were observed, including white matter signal abnormalities (19/69), major dilated Virchow–Robin spaces (12/69) and temporal lobe abnormalities (20/69). In all, 52% of the MRIs were interpreted as normal (36/69 patients).

**Conclusions:**

An unexpectedly high rate of MRI abnormalities was found in the first large series of clinical MRI investigations in non-syndromic autism. These results could contribute to further etiopathogenetic research into autism.

## Introduction

Autism is a neurodevelopmental disorder with a range of clinical presentations, from mild to severe, that is now classified in a broader class of disease called “autism spectrum disorders” (ASD). The most common clinical ASD sign is impaired social interaction, which is associated with verbal and non-verbal communication deficits and stereotyped behaviors [2]. In most cases, it is not presently possible to detect a known or specific etiology; these are referred to as non-syndromic autism [3]. The clinical relevance of MR scanning in children with ASD is still an open question and must be considered in light of the evolution of this technology. In 2000, MRI was judged to be of insufficient value to be included in the standard clinical evaluation of autism according to the guidelines of the American Academy of Neurology and Child Neurology Society [1]. This consensus stated that the prevalence of lesions detected by MRI in children with autism has been reported to be similar to normal control subjects [4]. However, this statement was based on results obtained from small samples of patients and, more importantly, included mostly insufficient MRI sequences. An adequate brain MRI interpretation must include at least three different sequences (T1, T2, FLAIR) in three different planes. Yet, there are few clinical radiological studies with complete clinical MRI examinations in children with ASD. For example, in some small groups of children with ASD, some radiological MRI anomalies were described, such as accentuated Virchow–Robin space [5], acrocallosal syndrome [6], pachygyria [7] macrogyria and polymicrogyria [8], [9]. However, until now, no reliable data has been available regarding the prevalence of MRI abnormalities in a large sample of patients with non-syndromic ASD.

Therefore, the aim of the present study was to evaluate the prevalence of brain abnormalities in a large group of children with non-syndromic autistic disorder (AD) using T1, T2 and FLAIR MRI sequences. In addition, in order to determine if the MRI abnormalities detected in the present population of children with non-syndromic AD could be also observed in a normal population of children, the same MRI clinical review was performed in 77 age-matched children without either developmental or neurological disorders.

## Methods

### Participants

Seventy-seven children and adolescents with AD (mean age±sd: 7.4±3.6, age range: 2.3 years–16,6 years; 64 boys) were studied as part of a series of imaging research projects focusing on non-syndromic AD. Children with AD were recruited in three university hospitals with dedicated units labeled as reference centers for autism by the French Health Ministry. This group of children was composed only of children with autistic disorder (AD). Children with Asperger syndrome or pervasive developmental disorder –not otherwise specified (PDD-NOS) were not included in this study. The inclusion criteria were age (range: 2–17 years) and non-syndromic AD diagnosis according to the DSM-IV and ADI-R [10] criteria for autism. Diagnosis was performed in these units by a multidisciplinary team including child psychiatrists, child psychologists and speech therapists during 3–7 days of extensive evaluation. The exclusion criteria were: 1) IQ below 40, 2) known infectious, metabolic or genetic diseases, 3) chromosomal abnormalities, 4) seizures, 5) identifiable neurological syndromes or focal neurological signs, 6) significant sensory impairment (e.g., blindness, deafness) or 7) major physical abnormalities.

All children were evaluated by a pediatric neurologist, a clinical geneticist and a child psychiatrist. In addition, the recommended biological and medical screenings for ASD were performed, including high-resolution karyotyping, DNA analysis of FRA-X and normal standard metabolic testing (plasma and urine amino and organic acid analysis, urine glycosaminoglycans (GAG) quantitation, urine oligosaccharide, purine and pyrimidine analysis, and creatine guanidoacetate urine analysis).

Mental retardation was assessed by an intelligence quotient (IQ), determined with the Wechsler Intelligence Scale for Children and the Wechsler Preschool and Primary Scale of Intelligence (WISC-III and WPPSI-III). DQ (Developmental Quotient), classic for assessment of mental retardation) was obtained in all children younger than 6 years old. Developmental quotient (DQ) was determined with the Psycho-Educational-Profile Revised (PEP-R) and the Brunet-Lézine developmental tests.

MRI inspection was also performed in 77 age-matched comparison children. The control MRIs were retrospectively selected from our database of children with cervico-facial pathologies (sinusitis, traumatism, facial dermoid cysts, facial cutaneous vascular malformations) and without neurological and neuro-chirurgical disorders according to three criteria: 1) age matching with AD children group, 2) MRI including all sequences performed in AD children and 3) clinical files providing sufficient information to ensure the lack of neurological or developmental disorders.

### Ethics

An Ethics Committee approved the study, and all examinations of autistic children were performed with the written informed parental consent.

The parents of control children did not provide explicit informed consent, since the French ethical legislation specifies that informed consent is automatically waived for retrospective studies using patient files.

### Description of procedure

#### Brain Imaging: T1,T2 and FLAIR

MRI was performed with a 1.5 Tesla (Signa General Electric) scanner using the following sequences: 3D T1-weighted FSPGR sequence (TR/TE/TI/NEX: 10.5/2.2/600/1, flip angle 10°, matrix size 256×192, yielding 124 axial slices and a thickness of 1.2 mm, field of view 22 cm), axial and coronal FSE T2-weighted imaging (TR/TE: 6000/120, 4 mm slices, 0.5 mm gap, field of view 22 cm) and coronal FLAIR sequences (TR/TETI: 10000/150/2250, 4 mm slices, 1 mm gap, field of view 24 cm). MRI studies were performed during sleep induced by premedication (7 mg/kg of sodium pentobarbital) for all AD children to obtain immobility during scans.

Signal intensities on T1, T2, and proton density-weighted images relate to specific tissue characteristics. For example, the changing chemistry and physical structure of hematomas over time directly affects the signal intensity on MR images, providing information about the age of the haemorrhage. The most common pulse sequences are the T1- weighted and T2-weighted spin-echo sequences. The T1-weighted sequence uses a short TR and short TE (TR<1000msec, TE<30msec). The T2-weighted sequence uses a long TR and long TE (TR>2000msec, TE>80msec). The T2-weighted sequence can be employed as a dual echo sequence. The first or shorter echo (TE<30msec) is proton density (PD) weighted or a mixture of T1 and T2. This image is very helpful for evaluating periventricular pathology, such as multiple sclerosis, because the hyperintense plaques are contrasted against the lower signal CSF. More recently, the FLAIR (Fluid Attenuated Inversion Recovery) sequence has replaced the PD image. FLAIR images are T2-weighted with the CSF signal suppressed. When reviewing an MR image, the easiest way to determine which pulse sequence was used, or the “weighting” of the image, is to look at the cerebrospinal fluid (CSF). If the CSF is bright (high signal), then it must be a T2-weighted imaged. If the CSF is dark, it is a T1-weighted or FLAIR image. Pathologic lesions can be separated into 4 major groups (solid mass, cyst, blood, fat) by their specific signal characteristics on the three basic images: T2- weighted, FLAIR, and T1-weighted. Since studies have shown that T2-weighted images are most sensitive for detecting brain pathology, patients with suspected intracranial disease should be screened with T2-weighted spin-echo and FLAIR images. T1-weighted images are needed only if the preliminary scans suggest hemorrhage, lipoma, or dermoid. The axial plane is commonly used because of the familiarity with the anatomy from CT. Coronal views are good for parasagittal lesions near the vertex and lesions immediately above or below the lateral ventricles, temporal lobes, sella, and internal auditory canals. The coronal plane can be used as the primary plane of imaging in patients with temporal lobe seizures. Sagittal views are useful for midline lesions (sella, third ventricle, corpus callosum, pineal region), and for the brainstem and cerebellar vermis.

### MRI criteria

MRI scans were interpreted independently by two board-certified radiologists experienced in pediatric neuroradiology (NB, FB – blinded for the diagnosis). Structural malformations or signal abnormalities of gray and white matter were rated for each examination. White matter abnormalities were classified as multiple punctate or plaque-like confluent hypersignals on T2 and FLAIR sequences. Dilated Virchow-Robin (VR) spaces were described as abnormal when they were >3 mm, which is consistent with the classification system developed by Heier et al [11]. These spaces are fluid-containing dilatations of the perivascular space that surround penetrating arteries in the brain and are characterized by smooth margins and isointensity to cerebrospinal fluid (CSF) on T1-weighted, T2-weighted and FLAIR MR images. Other structural malformations or signal abnormalities were described qualitatively. However, normal variants or minor abnormalities, such as posterior fossa cysts, abnormal hippocampal shape, ventricular dilatation or minor cerebellar atrophy, were not considered as abnormalities and were classified as normal MRIs [12]. All MRI sequences were also evaluated for the stage and pattern of myelination. MRI was considered to be uninterpretable when movement artifacts significantly degraded image quality.

### Statistical Methods

All statistical analyses were conducted using the SPSS statistical package (version 11.5). Categorization of the consensus is based on the method described by Landis and Koch [13]. The consensus for the radiologists' findings was 0.82 (Cohen's kappa, 95% CI = 0.72 to 0.93), which is considered to be very good. When there was a disagreement between the radiologists, the abnormality was not quoted.

## Results

### Participants

The group of children with AD was composed of 64 boys and 13 girls; their mean age was 7.4 years (SD = 3.6) and age ranged from 2.3 years to 16.6 years. The mean ADI-R score and sub scores are reported in Table 1. DQ was obtained in all children younger than 6 years old (n = 36). The mean global intelligence quotient (IQ or DQ) value was 60 (SD = 14) (Table 2). 30% of the children with AD had normal IQ or DQ (23 children).

**Table 1:** Clinical characteristics of the group of 77 children with autistic disorder.

	Mean	SD	Range	Threshold for AD diagnosis
ADI-R Global Score	52.6	12.9	24–75	
ADI-R Social Score	26.8	7.4	10–39	>10
ADI-R Non-Verbal Score	12.9	4.1	7–19	>7
ADI-R Communication Score*	20.4	4.6	8–30	>8
ADI-R Stereotypy Score	7.6	3.6	3–18	>3
ADI-R Onset Score	4.1	1.1	1–5	>1

ADI-R = Autism Diagnosis Interview – Revised

* Scores obtained in 42 children with sufficient verbal capacities

**Table 2:** Clinical and sociodemographic characteristics of children with AD related to normal and abnormal MRIs.

	Normal MRI (n = 36)	Abnormal MRI (n = 33)	P
Age	6.9 (SD = 3.4)	6.2 (SD = 1.9)	0.10
Sex ratio (% male)	77%	78%	
ADI-R Global Score	47.8 (SD = 10.4)	50.9 (SD = 10.9)	0.17
Mean IQ/DQ	61.1 (SD = 17.5)	54.6 (SD = 12.3)	0.09

The mean age of the comparison children (50 boys and 27 girls) was 7.0 years (SD = 4.2), with an age range between 2.4 and 18 years.

### MRI data

MRI was considered to be uninterpretable in 10% (8/77) of cases. MRI was rated as abnormal in 33/69 patients (40%), and three main types of abnormalities were found: white matter signal abnormalities (n = 19), severely dilated Virchow–Robin spaces (n = 12) and temporal lobe structural abnormalities (n = 20). As shown in Table 3, these abnormalities occurred in combination in 17 patients (25%). These abnormalities were never observed in our control group.

**Table 3:** Results of Brain MRI abnormalities in patients with non-syndromic Autism Spectrum Disorder.

	White Matter		Virchow-Robin	Temporal			Corpus Callosum	Heterotopia
	Punctate	Posterior	Dilated	Loss of gray/white matter definition	T2 sub- cortical hyper signal	Mass	Thick	
Isolated (n = 13)		2	6		3	1	0	1
Associated (n = 20)								
**n = 2**		X		X				
**n = 6**		X		X	X			
**n = 1**	X				X			
**n = 2**		X	X					
**n = 2**		X	X		X			
**n = 2**		X	X	X	X			
**n = 2**		X					X	
**n = 3**				X	X			

Fifty-two percent of the MRI scans in the AD group were considered to be normal (36/69 patients).

Clinical and demographic characteristics (age, sex, IQ and ADI scores) of AD children having normal or abnormal MRI were not significantly different (Table 2).

### Radiological description of the main abnormalities

**Figure 1:**
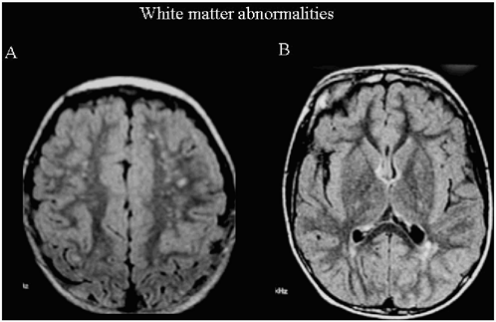
White matter abnormalities in autism. Two children illustrating the principal categories of white matter signal abnormalities. Figure 1A. Punctate T2 Hyperintensity: Abnormal findings were placed in this category when small (<2 mm) rounded abnormalities were found scattered bilaterally in the white matter (white arrow). They were asymmetric and homogeneous, and no findings suggest that necrosis was present. They were very intense compared with adjacent white matter on T2 and FLAIR sequences, and did not involve the basal ganglia, the periventricular white matter fibers or the sub-cortical U fibers. These abnormalities were generally found in association with other supratentorial abnormalities. Figure 1B. Posterior T2 Hyperintensity. Abnormalities placed in this category were “plaque-like areas” of mild white matter hyperintensity relatively symmetrical bilaterally at the posterior horns of the lateral ventricles (black arrow). There was no deformation of the lateral ventricular contour adjacent to these lesions. No abnormality of the sub-cortical U fibers was observed.

The white matter signal abnormalities (Figure 1 and Table 3) were mainly posterior hyperintensity “plaques-like areas” on T2, which were found in 19 patients (28%). They were relatively symmetrical and were located bilaterally at the posterior horns of the lateral ventricles, without deformation of the adjacent lateral ventricular wall and without involvement of the sub-cortical U fibers. White matter abnormalities were associated with other abnormalities in 17 patients.

**Figure 2:**
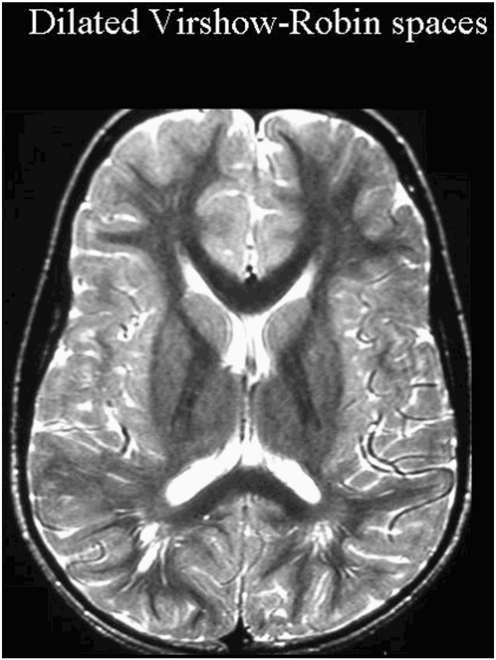
Dilated Virchow-Robin spaces in autism. Virchow-Robin (VR) spaces are fluid-containing dilatations of the perivascular space that surrounds penetrating arteries in the brain (white arrow). We defined abnormal VR spaces when the spaces were >3 mm using the classification system developed by Heier et al [11].

In addition, dilated VR spaces were found in 12 patients and were isolated in six patients (Figure 2 and Table 3). Enlarged VR spaces were characterized by smooth margins and a rounded or linear shape that conformed to the path of penetrating arteries, and were isointense to cerebrospinal fluid (CSF) on both T1-weighted and T2-weighted and FLAIR magnetic resonance (MR) images.

**Figure 3:**
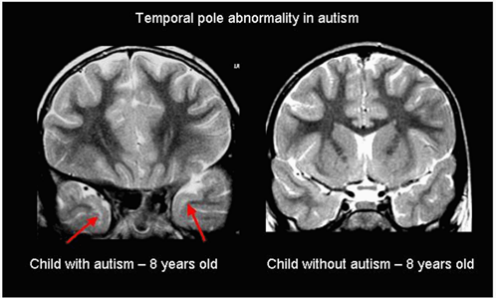
Temporal pole abnormalities in a child with autism. Example of a typical sub-cortical hyperintensities on T2-weighted coronal images localized in the temporal poles observed in children with autism (red arrows) and a normal image of a control child without autism.

Other structural abnormalities were detected only in the temporal lobes (Figure 3 and Table 3). They were found in 20 patients and consisted of sub-cortical hyperintensity on T2-weighted images localized in the temporal poles (n = 17, isolated in three patients) (Fig 3), loss of gray-white matter definition in the temporal poles on FLAIR-weighted images (n = 13, never isolated) and a unilateral nodular temporal lobe mass (n = 1). Sub-cortical temporal pole hyperintensities are usually observed during normal myelination (until four years of age); therefore, we only considered them as abnormal in children older than four years of age.

Concerning age and cognitive level, regarding all types of abnormalities there was no significant differences between the group with MRI abnormalities comparing to the group without MRI abnormalities for both age and IQ data. More detailed age analysis showed that in children younger than 4 years, MRI was abnormal in 4 children and normal in 6; from 4–6 years, MRI was abnormal in 12 children and normal in 16 children; from 6–8 years, MRI was abnormal in 10 children and normal in 3 children; from 8–10 years: MRI was abnormal in 7 children and normal in 4 children; MRI was normal in all children older than 10 years old (n = 7). In addition, within the MRI abnormalities, we found some interesting trends. For example, white matter abnormalities are more linked to young children than older (mean age with white matter abnormalities: 5.1±1.3 years old vs. children without white matter abnormalities 7.7±1.4 years old; p<0.01). One explanation of this age difference could be a delayed myelination. This was not the case for temporal lobe abnormalities (mean age with temporal abnormalities: 6.2±1.7 years old vs. children without temporal abnormalities 6.5±2.2 years old; p = 0.2).

Concerning IQ, temporal abnormalities are more linked to children with higher IQ (mean IQ with temporal abnormalities: 59.3±12.8 vs. children without temporal abnormalities 46.3±7.1; p<0.03). For white matter abnormalities there was no significant difference (mean IQ with white matter abnormalities: 57±13.4 old vs. children without white matter abnormalities 51.6±11.4).

Concerning the ADI sub-scores, the median social score (B) was 29. We found 5 children with temporal abnormalities with B score inferior than 29 and 11 children with B score superior than 29. Three children with white matter abnormalities had B score inferior than 29 and 8 children had B score superior than 29. For communication score (C), the median score was 13.5. We found 6 children with temporal abnormalities with C Score inferior than 13.5 and 10 superior than 13.5. Concerning white matter abnormalities, 5 children had a C score inferior to the median score and 6 superior to the median. So, the autistic children with more severe social deficits and language impairments had more temporal abnormalities.

## Discussion

To our knowledge, the present retrospective study reports the largest series of systematic visual analyses of MRI from patients with non-syndromic AD. These patients have been carefully screened to exclude known medical disorders associated with autism. We observed an unexpectedly high prevalence of brain abnormalities (48%). This unexpectedly high level of anomalies contrasts with the generally accepted view that MRI is close to normal in children with AD [1]. This could be explained by a methodological improvement, including here, of considering MRIs containing all the acquisitions necessary to detect brain abnormalities. We found three types of brain anomalies, including white matter hyperintensity on T2/FLAIR sequences, temporal lobe signal abnormalities and dilated Virchow-Robin spaces. Such abnormalities were not found in any child in the comparison group, which is in agreement with a recent MR study in a large group of normal children [14]. These abnormalities cannot be detected when only a T1 sequence is acquired. It is important to note that this high prevalence of abnormalities was found despite a stringent definition for an abnormal MRI. Indeed, all minor anomalies or normal variants (ventricular dilatation, accentuated Virchow-Robin spaces, abnormal hippocampal shape, arachnoid cysts, cerebellar atrophy, etc.) were not considered as abnormal. Similar results were found in a recent study that included a smaller sample of children with developmental disorders, including ASD, with abnormal MRI being reported in 49% of patients [9]. In addition, Taber et al. have also described a high incidence of abnormal Virchow-Robin spaces in children and adolescents with ASD and normal IQ [5].

Our study was subject to a number of limitations. One intrinsic limitation is that the comparison group was not matched for IQ with the AD group, which was largely composed of children with AD and mental retardation. Therefore, we cannot say whether these MRI abnormalities are specific to autism. Nevertheless, in our series, the 23 patients with normal IQ had the same types of MRI abnormalities as did patients with AD and mental retardation. In idiopathic mentally retarded children, the most frequently reported MRI abnormalities are ventricular dilatation, arachnoid cysts, moderate subarachnoid space enlargement, cerebellar atrophy and/or cortical atrophy, partially opened septum pellucidum and/or cavum vergae and corpus callosum anomalies [15], [16]. These types of abnormalities are often considered to be minor MRI findings and were not reported as abnormal in the present study. Nevertheless, they were rarely observed in the AD group (3%). Another limitation is that our findings cannot be extended to persons with high-functioning AD or to the full spectrum of ASD, which covers very heterogeneous disorders. Therefore, further clinical MRI investigations are necessary in these sub-groups of patients. Finally, another important issue will be to further characterize putative clinico-radiological sub-groups in AD and future studies need to be performed.

Certainly, the MRI abnormalities recognized in the present study are not specific to AD, since they have been previously reported in other neurological, metabolic or genetic childhood disorders. Posterior periventricular hyperintensity was found as a white matter signal abnormality in 18/77of the patients. Classically, this abnormality can be found in periventricular leukomalacia, metabolic disorders, viral infections or vascular disorders [17]. White matter MRI abnormalities were recently described in a large series of patients with cerebral palsy and were categorized into three levels of severity from mild to severe; in this study the abnormalities were always linked to motor deficits [18]. The white matter abnormalities that we have found in children with autism are comparable to the mild to moderate levels described in cerebral palsy, but no motor deficits were observed in our AD patients. Isolated or associated white matter abnormalities were found in 30/77 children with autism in our series. They could represent injury to the brain parenchyma and resultant disruption of neural circuitry. The main question is which different mechanisms may be involved in the emergence of such white-matter abnormalities. It is highly possible that these white matter hyperintensities (WMH) might simply represent the ‘tip of the iceberg’ in terms of structural white matter lesions. Thus, the presence and severity of white-matter hyperintensities associated with autism might be understood as an extreme consequence of underlying microstructural processes that affect brain connectivity and which may be more specifically investigated using diffusion tensor imaging methods. WMH, depending on the localization, are commonly classified as periventricular hyperintensities (PVH) or deep white matter hyperintensities (DWMH). Deep white matter hyperintensities were identified as having mainly a vascular etiology, and periventricular hyperintensities could be due to ependymal loss, differing degrees of myelination and cerebral ischemia. WMH are reported to be commonly associated with older age, and cardiovascular risk factors such as hypertension and diabetes. Lesions in one specific part or disruption of interconnections among areas regulating social and communication cognition could trigger the onset of autistic symptoms. Furthermore, posterior white matter connections with the temporal regions could be of particular importance to social disturbances in autism. Although, we did not measure white matter connections, lesions in such neuroanatomic pathways may be causal factors of behavioral and emotional dysfunctions in autistic patients. Finally, it is also important to understand how WMH severity changes over time.

In our series, dilated Virchow- Robin spaces were demonstrated in 12/77 patients. Classically, these abnormalities have been found in muccopolysaccharidosis [19]. Temporal lobe abnormalities were characterized mainly by white matter signal abnormalities. These types of white matter abnormalities are frequently described in childhood disorders such as epilepsy and in congenital cytomegalovirus infection [20]. Because a sub-cortical hypersignal on T2 and FLAIR sequences localized in the temporal poles is a normal finding in normal children under four years of age, the persistent sub-cortical hypersignal within the temporal poles could represent an immature pattern of myelination. Therefore, further detailed pediatric neurology consultation and sleeping EEG investigations could be useful in such cases.

The detection of unexpected MRI abnormalities in children with AD could have at least one positive impact, allowing for further etiopathological investigations that are not suggested by classical clinical and biological assessments. The MRI abnormalities observed in children with autism in the present manuscript are non specific to autism but specificity of MRI abnormalities concerns a large few of neurological disorders. In only few clinical situation, radiological patterns of MRI abnormalities lead to specific etiopathological diagnosis (for example, a bilateral hemorrhagic necrosis temporal lobe in Herpes simplex virus). It has been generally assumed that the specificity of MRI is much lower than its sensitivity. In most cases of neuropediatrics disorders, on the basis of similarities in the pathogenesis, a similar clinical picture and similar MR images, the type and the localization of MRI abnormalities guides further etiopathological investigations facilitating the diagnosis process and reducing the list of necessary laboratory tests [21]. Structural elements that have been identified as having in general the highest discriminating value are: symmetry versus asymmetry, confluent involvement of the white matter versus multifocal isolated lesions, the predominant localization of lesions in the brain and the additional involvement of gray matter structures. For example, symmetry is a striking feature of inherited white matter disorders and toxic encephalopathies, whereas asymmetry is most often seen in acquired white matter disorders, particularly inflammatory disorders and infections. The distribution of the lesion may give an indication of the type of disorder we are dealing with. Sparing of the U fibers is seen in many inherited white matter disorders and in vascular disorders. Several organic and amino acidopathies, contrariwise, preferentially involve the U fibers. So, the pattern emerging from the image can be expressed as being diagnostic (pathognomonic), highly suggestive, suggestive, possible, atypical, and impossible. It is only in the first category that the MRI pattern is pathognomonic for one specific disorder. In the other categories, clinical and laboratory evidence is necessary to complete the diagnosis. The MRI abnormalities observed in children with autism in the present manuscript are therefore non specific, but they can guide to more focus etiological investigations. In our series, an association between at least two abnormalities was found in 26% of the patients (20/77 patients). In most of these cases, temporal lobe abnormalities were associated with white matter hyperintensities. Such unusual association of MRI abnormalities is, to our knowledge, not linked to any specific pediatric pathology. Therefore, further more specific genetic and metabolic studies are needed to better understand the relationship between these associated temporal anomalies and white matter hyperintensity in autism. MRI findings could be helpful to constitute a more homogeneous sub-grouping of patients with AD. These radiologically-based sub-groups could be useful for genetic research.

Indeed, endophenotypes represent intermediate phenotypes on the putative causal pathway from the genotype to the phenotype. They offer a potentially valuable strategy to examine the molecular etiopathology of complex behavioral phenotypes such as autism. Few studies have examined the structural variations in the brain that might underlie the functional impairments as useful endophenotypes for autism. Over the past three decades, there has been an impressive body of literature supporting brain structural alterations in autism. The role of genetic factors in the etiology of autism has been supported by a high heritability for autism. This suggests that a large part of the variance for the etiology of autism is contributed by genetic factors. Having stated this, the precise nature of genetic basis of autism still remains unclear. It is still unclear regarding which gene or genes are responsible and variations in how many genes are required for the onset of the clinical syndrome. The poorly defined phenotypic boundaries, genetic investigations have been hampered by small effect of the identified genetic risk loci, poorly understood pathophysiology, possible contribution of several genes, and complex interactions among the genes and with the contributing environmental factors. Another important advantage to examining the endophenotypes is that they are quantifiable as continuous measures, and therefore, a dimensional approach is tenable while employing endophenotypes as one of the outcome variables. The found MRI abnormalities could help to further distinguish sub-groups in autism and therefore could constitute a first step in the construction of brain imaging endophenotypes in autism.

The present study contrasts with the main literature of brain imaging studies in ASD that mostly searched for quantitative or functional brain abnormalities. These previous studies do not provide qualitative clinical radiological information, mainly because all appropriate clinical MRI sequences are not usually performed for such purposes. They involve quantitative analyses such as region of interest or voxel by voxel strategies performed mostly on 3D-T1 MRI sequence (for review see [22]). The most compelling structural findings are an increase of the total brain volume. Furthermore, a number of brain imaging studies in ASD have found anatomical and functional abnormalities in regions belonging to social-cognitive neural networks (superior temporal sulcus, face-fusiform area, amygdala, mirror-neuron system, frontal and parietal cortex) (for review see [23]). Interestingly, these quantitative brain abnormalities included the temporal lobes, the region in which we have found abnormalities, suggesting a possible link between quantitative and qualitative approaches.

Further clinical MRI studies should include a 1H MRS magnetic resonance spectroscopy sequence since recent publications have described creatine deficiencies in autistic disorders that were linked to known genetic anomalies [24], [25].

In conclusion, an unexpectedly high rate of MRI abnormalities was found in the first large series of clinical MRI investigations in children with non-syndromic autism. These results could contribute to further etiopathogenetic research into autism.
